# Daily omega-3 fatty acid intake and depression in Japanese patients with newly diagnosed lung cancer

**DOI:** 10.1038/sj.bjc.6601621

**Published:** 2004-02-17

**Authors:** S Suzuki, T Akechi, M Kobayashi, K Taniguchi, K Goto, S Sasaki, S Tsugane, Y Nishiwaki, H Miyaoka, Y Uchitomi

**Affiliations:** 1 Psycho-Oncology Division, National Cancer Center Research Institute East, 6-5-1 Kashiwanoha, Kashiwa, Chiba 277-8577, Japan; 2 Department of Psychiatry, Kitasato University, 1-15-1 Kitasato, Sagamihara, Kanagawa 228-8555, Japan; 3 Epidemiology and Biostatistics Division, National Cancer Center Research Institute East, 6-5-1 Kashiwanoha, Kashiwa, Chiba 277-8577, Japan; 4 Thoracic Oncology Division, National Cancer Center Hospital East, 6-5-1 Kashiwanoha, Kashiwa, Chiba 277-8577, Japan; 5 National Institute of Health and Nutrition, 1-23-1 Toyama, Shinjuku-ku, Tokyo 162-8636, Japan

**Keywords:** omega-3 fatty acid, *α*-linolenic acid, depression, lung cancer, cross-sectional study

## Abstract

The aim of the present study was to examine the association between daily omega-3 fatty acid intake and depression in Japanese cancer patients. Omega-3 fatty acid intake in 771 patients with newly diagnosed primary lung cancer was evaluated using a food-frequency questionnaire, and the prevalence of depression was examined using the cutoff values for the depression subscale included in the Hospital Anxiety and Depression Scale. After adjustment for potential confounding factors, the odds ratio (OR) for depression among patients in the highest quartile of the total eicosapentaenoic acid- (C20:5*n*-3) and docosapentaenoic acid (C22:6n-3)-intake group compared with patients in the lowest quartile was not significantly different. On the other hand, the OR among the highest quartile of *α*-linolenic acid (C18:3n-3) intake (adjusted OR=0.50, 95% CI: 0.31–0.71, *P* for trend=0.004) and the highest quartile of total omega-3 fatty acid intake (adjusted OR=0.55, 95% CI: 0.35–0.88, *P* for trend=0.022) were significantly different. These results suggest that total eicosapentaenoic acid and docosapentaenoic acid intake might not be associated with depression in Japanese patients with newly diagnosed lung cancer, but that *α*-linolenic acid intake and total omega-3 fatty acid intake might be.

Depression is the most common psychiatric problem in clinical oncology settings, with around 20% of cancer patients experiencing depression (McDaniel *et al*, 1995; Massie and Popkin, 1998). Depression influences the quality of life of cancer patients and often leads to a poor compliance with cancer treatment and suicidal ideation or suicide (Grassi *et al*, 1996; Breitbart *et al*, 2000; Colleoni *et al*, 2000; Akechi *et al*, 2002). Antidepressants are the only well-established treatment for depression in cancer patients (Gill and Hatcher, 1999); however, antidepressant therapy is difficult to perform in cancer patients, because the adverse effects of antidepressants might overload the patient's physical condition (Akizuki *et al*, 2002). Thus, a novel medication with few adverse effects is needed for the management of depression in cancer patients.

Recent psychiatry studies have investigated the association between depression and omega-3 fatty acids (*ω*-3 FAs), a potentially natural and tolerable antidepressant. Omega-3 fatty acids, which have a double bond at the third carbon from the methyl end of the molecule, include eicosapentaenoic acid (EPA; C20:5n-3) and docosahexaenoic acid (DHA; 22:6n-3), which are derived from fish and seafood, and *α*-linolenic acid (*α*-LA; C18:3n-3), which is derived from plant sources. Humans lack the desaturase enzyme, so *ω*-3 FAs cannot be formed in the body; thus, dietary intake is the only source of *ω*-3 FAs. The depletion of cell membrane *ω*-3 FAs has been hypothesised to be of aetiologic importance in depression (Smith, 1991; Hibbeln and Salem, 1995; Maes and Smith, 1998).

Some clinical evidence has supported the hypothesis that low levels of *ω*-3 FAs may be implicated in depression. Epidemiological findings linking depression and low levels of fish intake have been reported (Hibbeln, 1998; Tanskanen *et al*, 2001), and biochemical research has also shown that the concentrations of each *ω*-3 FA are significantly lower in the serum or red blood cell membranes of depressed patients than in control subjects (Edwards *et al*, 1998; Peet *et al*, 1998; Maes *et al*, 1999). Ethyl-EPA augmentation therapy has been reported to improve depressive symptoms in patients with major depression in randomised placebo-controlled double-blind clinical trials (Nemets *et al*, 2002; Peet and Horrobin, 2002) and in one clinical report (Puri *et al*, 2002). These trials have also reported no or a few adverse effects of the medication (e.g. mild gastrointestinal tract distress). In addition, the antidepressive effect of *ω*-3 FAs has also been reported in patients with other psychiatric conditions, including depression in pregnant woman (Chiu *et al*, 2003), borderline personality disorder (Zanarini and Frankenburg, 2003), and bipolar disorder (Stoll *et al*, 1999). This clinical evidence suggests that the administration of *ω*-3 FAs, especially EPA or DHA, may be useful for the management of depression in various situations.

As a preliminary examination of the utility of administering *ω*-3 FAs for the treatment or prevention of depression in cancer patients, we performed a cross-sectional study on the relationship between the daily intake of each *ω*-3 FA (as calculated using a food-frequency questionnaire (FFQ)) and the prevalence of depression in newly diagnosed Japanese patients with lung cancer. We hypothesised that a high intake of EPA and DHA may be associated with a low prevalence of depression.

## METHODS

### Subjects

This study was approved by the Institutional Review Board and the Ethics Committee of the National Cancer Center, Japan.

Data from subjects who participated in The Lung Cancer Database Project at the National Cancer Center Hospital East and the National Cancer Center Research Institute East were used in the present study. The aim of The Lung Cancer Database Project is to construct a comprehensive database of information that can be used to investigate the pathogenesis of lung cancer and improve currently available treatments. The database includes information on demographic factors, physical symptoms, psychological factors, and lifestyle factors (diet, smoking, etc) obtained from self-reported questionnaires and medical information from the patients’ medical charts and blood, DNA, and urine specimens.

The subjects enrolled in the present study were all newly diagnosed patients with primary lung cancer, who visited the Thoracic Oncology Division of the National Cancer Center Hospital East, Japan. Patients were included in this database study if they met all of the following criteria: informed of their lung cancer diagnosis; lung cancer diagnosis confirmed by histological examination; physically capable of completing the questionnaires; absence of cognitive impairment, such as dementia and delirium; ability to provide written consent; no problems regarding the patients’ participation in this database study, as judged by their physicians.

### Study procedure

Between July 1999 and November 2001, consecutive patients eligible for The Lung Cancer Database Project were recruited after the disclosure of their diagnosis by their attending physician. The patients completed the questionnaires during the waiting period prior to admission, and the questionnaires were collected after the patients were admitted. All patients provided their written informed consent prior to enrolment in this project.

### Measurement of depression

We used the Hospital Anxiety and Depression Scale (HADS) (Zigmond and Snaith, 1983), which consists of a seven-item anxiety subscale and a seven-item depression subscale to assess the anxiety and depressive symptoms during the preceding week in medically ill patients. The HADS has been used as a reliable and valid method of screening for depression in patients with cancer (Razavi *et al*, 1990; Hopwood *et al*, 1991; Patrick *et al*, 2003). Each item is rated on a scale of 0–3, with higher scores denoting a greater mood disturbance. The reliability and validity of the Japanese version of this questionnaire has been established in Japanese cancer patients (Kugaya *et al*, 1998). In this study, depression was evaluated using the depression subscale (HADS-D) with a cutoff point of 4/5, since this cutoff point has been found to yield a good sensitivity and specificity (91.5 and 58.0%, respectively) (Kugaya *et al*, 1998) for the screening of depression (adjustment disorder and major depression).

### Assessment ω-3 FA intake

The semiquantitative FFQ was made for a population-based prospective study in Japan (Tsubono *et al*, 1996), and contains questions regarding 138 foods (including 18 fish and seafood items). For each food item, the participants reported the average frequency of consumption during the preceding year prior to the onset of their cancer symptoms and specified the usual serving size. Nine responses were possible for each food item, ranging from ‘never’ to ‘7 or more times per day’. The average daily intake of nutrients was calculated by multiplying the frequency of the consumption of each item by its nutrient content per serving and totalling the nutrient intake for all food items. The method used to calculate the average daily intake of each food and nutrient based on the FFQ responses has been described elsewhere (Sasaki *et al*, 2003).

The *ω*-3 FA family includes *α*-LA (C18:3n-3), octadecatetraenoic acid (C18:4n-3), eicosatetraenoic acid (C20:4n-3), EPA (C20:5n-3), docosapentaenoic acid (DPA; C22:5n-3), and DHA (22:6n-3). We calculated the intake of each of these compounds, the total EPA and DHA intake, and the total *ω*-3 FA intake. The Fatty Acid Composition Table of Japanese Foods (Science and Technology Agency of Japan, 1990) was used to calculate the daily intake of each *ω*-3 FA. Since the table has missing values for some foods, we substituted the fatty acid composition of the missing foods (Sasaki *et al*, 1999).

The validity of the FFQ was assessed in a random sample of 102 men and 113 women living in four areas of Japan by comparing the data from the FFQ with the data from 14–28 dietary records collected approximately 6 months apart. The Spearman rank correlation coefficients for an association between the data from the FFQ and the dietary records for the men and women were 0.26 (95% CI, 0.04–0.48) and 0.28 (0.13–0.44), for the total *ω*-3 FAs intake (energy-adjusted values according to energy density), respectively, 0.32 (0.16–0.48) and 0.20 (0.05–0.34) for *α*-LA intake; 0.40 (0.09-0.72) and 0.47 (0.34–0.60) for octadecatetraenoic acid intake; 0.27 (−0.06–0.6) and 0.30 (0.17–0.44) for eicosatetraenoic acid intake; 0.38 (0.09–0.67) and 0.41 (0.26–0.56) for EPA intake; 0.36 (0.05–0.67) and 0.35 (0.22–0.49) for DPA intake; and 0.34 (0.07–0.61) and 0.36 (0.21–0.51) for DHA intake.

### Assessment of demographical and medical factors

Demographic factors (age, gender, education level, employment status, marital status, smoking, and drinking habits) and medical factors (histology, clinical stage, and symptoms) were obtained from the self-reported questionnaires and the patients’ medical charts. Performance status (PS) was assessed by each attending physician using the Eastern Cooperative Oncology Croup criteria.

### Statistical analyses

Odds ratios were calculated by dividing the prevalence of depression in each of four equal quartile groups (grouped according to the intake of each *ω*-3 FA) by the prevalence of depression in the lowest quartile of *ω*-3 FA intake. The same analysis was conducted using quartiles grouped according to total *ω*-3 FA intake and total EPA and DHA intake. A logistic-regression analysis, with adjustments for sex and age in 10-year groupings, was then performed; simultaneously, pain (present/absent) and performance status (0/1–4) were used as factors well known to be strongly associated with depression in cancer patients, and smoking status (current-smoker/ex-smoker/nonsmoker) and amount of alcohol consumption were used as factors thought to influence *ω*-3 FA metabolism (Simon *et al*, 1996; Pawlosky and Salem, 1999). Some background variables that were significantly associated with depression in this sample, according to a bivariate analysis (*χ*^2^ test), were also included; histology (small/non-small-cell carcinoma), clinical stage (Ia-IIb/IIIa-IV), breathlessness (present/absent), and employment status (employed/unemployed) (Table 1

**Table 1:** Patients’ characteristics

		**HADS-D**^a^		
		**⩽4 (*n*=335)**	**⩾5 (*n*=436)**	
**Variable**		***n* (%)**	***n* (%)**	***P*-value**
Age (mean±s.d. years)		64.0 (9.5)	64.3 (9.1)	0.651
Sex	Female	93 (27.8)	122 (28.0)	0.946
Education	⩽9years	101 (30.1)	136 (31.2)	0.705
Marital status	Married	280 (83.6)	370 (84.9)	0.669
Employment status	Full & part time	151 (45.1)	152 (34.9)	0.004

*Histology*				
Adenocarcinoma		221 (66.0)	240 (55.0)	0.003
Squamous		67 (20.0)	87 (20.0)	
Small		20 (6.0)	57 (13.1)	
Large		21 (6.3)	36 (8.3)	
Others		6 (1.8)	16 (3.7)	

*Clinical stage*^b^				
Ia		111 (33.1)	72 (16.5)	0.000
Ib		59 (17.6)	64 (14.7)	
IIa		4 (1.2)	7 (1.6)	
IIb		15 (4.5)	28 (6.4)	
IIIa		19 (5.7)	42 (9.6)	
IIIb		49 (14.6)	93 (21.3)	
IV		78 (23.3)	130 (29.8)	

*Performance status*^c^				
0		168 (50.1)	141 (32.3)	0.000
1		161 (48.1)	261 (59.9)	
2		6 (1.8)	24 (5.5)	
3		0 (0.0)	10 (2.3)	
4		0 (0.0)	0 (0.0)	

*Symptoms*				
Breathlessness (presence)		102 (30.4)	226 (51.8)	0.000
Pain (presence)		86 (25.7)	176 (41.4)	0.000

*Smoking*				
Current smoker		179 (53.4)	244 (56.0)	0.444
Ex-smoker		66 (19.7)	91 (20.9)	
Nonsmoker		87 (26.0)	97 (22.2)	

Total energy intake (kcal)		1819±1534	1815±529	0.919
Total fat intake (% of energy)		26.2±6.8	25.6±6.7	0.192

*Omega-3 fatty acid intake (% of energy)*				
*α*-LA intake (C18:3n-3)		0.93±0.30	0.86±0.30	0.002
Octadecatetraenoic acid intake (C18:4n-3)		0.04±0.02	0.03±0.02	0.375
Eicosatetraenoic acid intake (C20:4n-3)		0.02±0.01	0.02±0.01	0.361
Eicosapentaenoic acid intake (C20:5n-3)		0.16±0.09	0.16±0.10	0.364
Docosapentaenoic acid intake (C22:5n-3)		0.04±0.02	0.04±0.03	0.459
Docosahexaenoic acid intake (C22:6n-3)		0.28±0.14	0.27±0.15	0.372
Docosahexaenoic acid and eicosapentaenoic acid intake		0.45±0.23	0.43±0.24	0.367
Total omega-3 fatty acid intake		1.52±0.47	1.43±0.48	0.013

a HADS-D: Depression subscale of the Hospital Anxiety and Depression Scale.

b Defined by TNM classification: International Union Against Cancer.

c Defined by the Eastern Cooperative Oncology Group.

). We also tested for significant trends by assigning each patient the median value of their category and modelling it as a continuous variable.

A *P*-value of <0.05 was considered to be statistically significant; all tests were two-tailed. All statistical analyses were performed on a personal computer using the statistical software package SPSS for Windows (Version 11.0J, SPSS Japan Institute Inc.).

## RESULTS

### Participants

In total, 1013 patients with newly diagnosed, untreated primary lung cancer were admitted during The Lung Cancer Database Project enrolment period. A total of 42 patients were ineligible, the most common reason for exclusion being that the patient's condition was too poor for the patient to participate in the project. Of the remaining 971 eligible patients, 30 refused to participate in this study and 39 could not be contacted (e.g., because of emergency admission and the immediate start of treatment). As a result, 902 patients were enrolled in the project. After excluding the patients who did not complete the HADS-D (*n*=73) or who did not answer the FFQ (*n*=62), or who provided inappropriate answers to the FFQ (a total energy intake of below 900 kcal day^−1^ or above 4000 kcal day^−1^ in men or below 800 kcal day^−1^ or above 3600 kcal day^−1^ in women, *n*=24), a total of 771 patients were eligible for analysis. No significant gender differences were observed between the subjects who were analysed (*n*=771 patients) and the subjects who were not (*n*=242 patients). The analysed subjects were younger than the subjects who were not analysed (*P*=0.010), their disease was in clinical stage Ia-IIb (*P*=0.007), and they had a better PS (*P*=0.004). Among the 771 analysed patients, total *ω*-3 FA intake was primarily from vegetable oils and fats (37% of the total intake) and secondarily from 17 types of fish (35%), soybean products (11%), seasonings (5%), and cereals (4%). The proportional daily intake of *ω*-3 FAs consisted of 62% *α*-LA, and 20% DHA and 11% EPA.

### Depression and ω-3 FA intake

Of the 771 patients analysed, 436 patients (56.5%). had depression (Table 1).

The crude odds ratio for depression among patients in the highest quartile of the total EPA and DHA intake was 0.82 (95% CI 0.54–1.22) compared with patents in the lowest quartile, and the adjusted odds ratio after controlling for possibly associated factors was 0.96 (95% CI 0.61–1.49); no significant trend in the odds ratios for depression was observed with regard to total EPA and DHA intake (*P* for trend=0.648, Table 2

**Table 2:** Odds ratio of depression according to quartiles of omega-3 fatty acids intake (% energy) by the lung cancer patients (*n*=771)

		**HADS-D**^a^		**Odds ratio**		
		**⩽4**	**⩾5**	**Crude**	**Adjusted for age and sex**	**Multivariate**^b^
**Median Intake (% energy)**		***n* (%)**		**(95% CI)^c^**		
*Quartiles of α-linolenic acid intake (C18:3n-3)*						
q^1^	0.552	68 (35.4)	124 (64.6)	1.00	1.00	1.00
q^2^	0.776	80 (41.5)	113 (58.5)	0.78 (0.51–1.17)	0.76 (0.50–1.15)	0.68 (0.43–1.07)
q^3^	0.982	93 (48.2)	100 (51.8)	0.59 (0.39–0.89)	0.56 (0.37–0.85)	0.55 (0.34–0.87)
q^4^	1.223	94 (48.7)	99 (51.3)	0.58 (0.38–0.87)	0.54 (0.35–0.82)	0.50 (0.31–0.71)

*P* for trend				0.004	0.002	0.004

*Quartiles of octadecatetraenoic acid intake (C18:4n-3)*						
q^1^	0.015	71 (40.0)	121 (60.0)	1.00	1.00	1.00
q^2^	0.024	83 (43.0)	110 (57.0)	0.78 (0.52–1.17)	0.78 (0.52–1.18)	0.72 (0.46–1.13)
q^3^	0.034	96 (49.7)	97 (50.3)	0.59 (0.40–0.89)	0.58 (0.39–0.88)	0.61 (0.40–0.95)
q^4^	0.062	85 (44.0)	108 (56.0)	0.75 (0.50–1.12)	0.74 (0.49–1.13)	0.83 (0.53–1.30)

*P* for trend				0.250	0.250	0.721

*Quartiles of eicosatetraenoic acid intake (C20:4n-3)*						
q^1^	0.008	75 (39.1)	117 (60.9)	1.00	1.00	1.00
q^2^	0.001	81 (42.0)	112 (58.0)	0.89 (0.59–1.33)	0.89 (0.59–1.34)	0.88 (0.57–1.36)
q^3^	0.017	97 (50.3)	96 (49.7)	0.63 (0.42–0.95)	0.64 (0.42–0.95)	0.63 (0.41–0.97)
q^4^	0.027	82 (42.5)	111 (57.5)	0.87 (0.58–1.30)	0.86 (0.57–1.30)	0.97 (0.62–1.51)

*P* for trend				0.434	0.409	0.864

*Quartiles of eicosapentaenoic acid intake (C20:5n-3)*						
q^1^	0.074	78 (40.6)	114 (59.4)	1.00	1.00	1.00
q^2^	0.115	77 (39.9)	116 (60.1)	1.03 (0.69–1.55)	1.05 (0.70–1.58)	0.99 (0.64–1.55)
q^3^	0.165	95 (49.2)	98 (50.8)	0.71 (0.47–1.06)	0.69 (0.46–1.04)	0.55 (0.42–1.01)
q^4^	0.265	85 (44.0)	108 (56.0)	0.87 (0.58–1.30)	0.86 (0.57–1.30)	0.99 (0.64–1.55)

*P* for trend				0.332	0.312	0.849

*Quartiles of docosapentaenoic acid intake (C22:5n-3)*						
q^1^	0.020	75 (39.1)	117 (60.9)	1.00	1.00	1.00
q^2^	0.032	82 (42.5)	111 (57.5)	0.87 (0.58–1.30)	0.88 (0.58–1.32)	0.88 (0.57–1.36)
q^3^	0.454	91 (47.2)	102 (52.8)	0.72 (0.48–1.01)	0.72 (0.48–1.08)	0.70 (0.45–1.08)
q^4^	0.704	87 (45.1)	106 (54.9)	0.78 (0.52–1.17)	0.77 (0.51–1.17)	0.88 (0.57–1.36)

*P* for trend				0.218	0.197	0.556

*Quartiles of docosahexaenoic acid intake (22:6n-3)*						
q^1^	0.140	74 (38.5)	118 (61.5)	1.00	1.00	1.00
q^2^	0.206	82 (42.5)	111 (57.5)	0.85 (0.57–1.28)	0.86 (0.57–1.30)	0.95 (0.61–1.47)
q^3^	0.290	93 (48.2)	100 (51.8)	0.67 (0.45–1.01)	0.67 (0.44–1.00)	0.60 (0.38–0.93)
q^4^	0.435	86 (44.6)	107 (55.4)	0.78 (0.52–1.17)	0.77 (0.51–1.17)	0.94 (0.60–1.47)

*P* for trend				0.216	0.199	0.584

*Quartiles of Total omega-3 fatly acid intake*						
q^1^	0.943	67 (34.9)	125 (65.1)	1.00	1.00	1.00
q^2^	1.275	87 (45.1)	106 (54.9)	0.65 (0.43–0.99)	0.63 (0.42–0.96)	0.63 (0.40–0.99)
q^3^	1.603	86 (44.6)	107 (55.4)	0.67 (0.44–1.01)	0.64 (0.42–0.97)	0.61 (0.38–0.97)
q^4^	2.007	95 (49.2)	98 (50.8)	0.55 (0.37–0.83)	0.52 (0.34–0.80)	0.55 (0.35–0.88)

*P* for trend				0.008	0.005	0.022

*Quartiles of sum of eicosapentaenoic and docosahexaenoic acid intake*						
q^1^	0.217	76 (39.6)	116 (60.4)	1.00	1.00	1.00
q^2^	0.322	79 (40.9)	114 (59.1)	0.95 (0.63–1.42)	0.96 (0.64–1.44)	1.00 (0.65–1.56)
q^3^	0.456	94 (48.7)	99 (51.3)	0.69 (0.46–1.03)	0.68 (0.45–1.02)	0.64 (0.41–0.98)
q^4^	0.700	86 (44.6)	107 (55.4)	0.82 (0.54–1.22)	0.81 (0.54–1.22)	0.96 (0.61–1.49)

*P* for trend				0.237	0.221	0.648

a HADS-D: Depression subscale of the Hospital Anxiety and Depression Scale.

b Adjusted for age, sex, performance status, clinical stage, histology, pain, breathlessness, employment, smoking, alcohol consumption, and body mass index as covariates.

c 95% confidence interval.

and Figure 1

**Figure 1:**
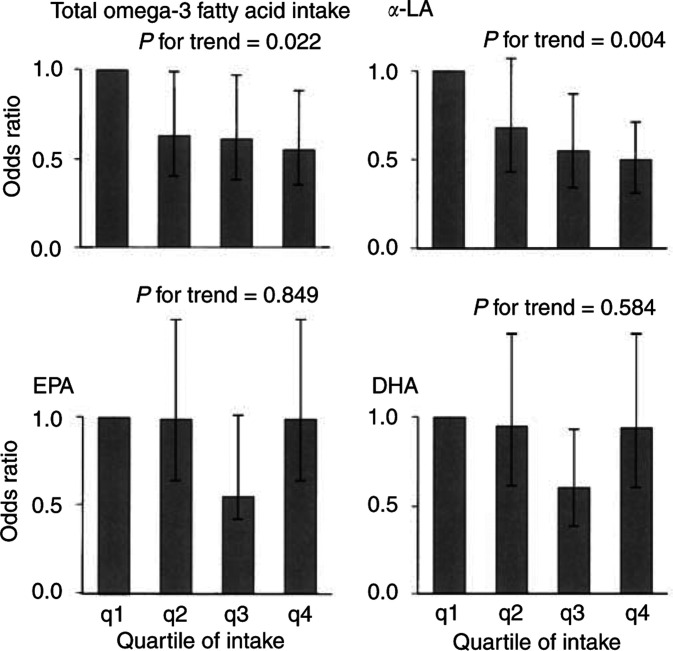
Odds ratio for depression in Japanese patients with newly diagnosed lung cancer categorised by quartile of *ω*-3 FAs intake (*n*=771). Adjusted for age, sex, performance status, clinical stage, histology, pain, breathlessness, employment, smoking, alcohol consumption, and body mass index. The widths of the bars represent 95% confidence intervals. Significant inverse relationship was observed between total *ω*-3 FA or *α*-linolenic acid and depression. *α*-LA, *α*-linolenic acid; EPA, eicosapentaenoic acid; DHA, docosahexaenoic acid.

). No significant negative association was found between either EPA intake or DHA intake and depression (EPA: adjusted odds ratio=0.99, 95% CI: 0.64–1.55, *P* for trend=0.849; DHA: adjusted odds ratio=0.94, 95% CI: 0.60–1.47, *P* for trend=0.584).

The crude odds ratio for depression among patients in the highest quartile of *α*-LA intake group was 0.58 (95% CI 0.38–0.87) compared with patents in the lowest quartile, and the adjusted odds ratio after controlling for the possibly associated factors was 0.50 (95% CI 0.31–0.71); a significant trend in the odds ratio for depression was observed with regard to total *ω*-3 FA intake (*P* for trend=0.004, Table 2 and Figure 1). A significant negative association was also found between total *ω*-3 FA intake and depression (crude odds ratio=0.55, 95% CI: 0.37–0.83, *P* for trend=0.008; adjusted odds ratio=0.55, 95% CI: 0.35–0.88, *P* for trend=0.022). In addition, we performed the same analysis for 786 subjects including subjects whose answers were regarded as indicating an extremely low caloric intake (below 900 kcal day^−1^ in men or below 800 kcal day^−1^ in women, *n*=15), since it was impossible to discount the possibility that those patients may have had a low caloric intake for a variety of reasons (e.g., chronic depression). The results of this analysis were similar to the previously obtained results (data not shown).

No association between depression and the intake of fish and seafood was observed (adjusted OR=0.81, 95% CI: 0.52–1.26, *P* for trend=0.285, Table 3

**Table 3:** Odds ratio of depression according to quartiles of fish intake (g per 1000 kcal) by the lung cancer patients (*n*=771)

		**HADS-D**^a^		**Odds ratio**		
		**⩽4**	**⩾5**	**Crude**	**Adjusted for age and sex**	**Multivariate**^b^
**Quartile**	**Median intake (g per 1000 kcal)**	***n* (%)**	***n* (%)**	**(95% Cl^c^)**		
q^1^	20.5	77 (40.1)	115 (59.9)	1.00	1.00	1.00
q^2^	32.3	80 (41.5)	113 (58.5)	0.95 (0.63–1.42)	0.94 (0.62–1.42)	0.96 (0.62–1.50)
q^3^	44.4	87 (45.1)	106 (54.9)	0.82 (0.54–1.22)	0.81 (0.54–1.21)	0.79 (0.51–1.23)
q^4^	67.0	91 (47.2)	102 (52.8)	0.75 (0.50–1.12)	0.73 (0.49–1.11)	0.81 (0.52–1.26)

*P* for trend				0.127	0.107	0.285

a HADS-D: Depression subscale of the Hospital Anxiety and Depression Scale.

b Adjusted for age, sex, performance status, clinical stage, histology, pain, breathlessness, employment, smoking, alcohol consumption, and body mass index as covariates.

c 95% confidence interval.

).

## DISCUSSION

This report is the first cross-sectional study to investigate the association between dietary *ω*-3 FA intake, as calculated using FFQ data, and the prevalence of depression among cancer patients. Inconsistent with the results of a previous epidemiological study, we did not observe a significant association between total DHA and EPA intake and the prevalence of depression in newly diagnosed patients with lung cancer. This finding suggests that DHA and EPA intake may not contribute to a susceptibility to depression among Japanese cancer patients. Since the standard Japanese diet contains larger amounts of fish than the diets of Europeans or Americans, the antidepressive effect of DHA and EPA may not be apparent in our sample. Another possible reason for the discrepancy in findings is that the characteristics of depression in this study may differ from those of previous studies. Previous epidemiological research examined the prevalence of depression in the general population (Hibbeln, 1998; Tanskanen *et al*, 2001), while we examined depression among a population that was subjected to the stress of having recently been diagnosed with lung cancer. Thus, differences in the situations of the subjects may have caused the inconsistent findings. In addition, our results must be interpreted with caution, because our sample size was smaller than that of previous epidemiological studies.

Interestingly, a significant inverse dose–response relationship was observed between *α*-LA or total *ω*-3 FA intake and the prevalence of depression. To the best of our knowledge, this is one of the first clinical reports to demonstrate an association between *α*-LA intake and depression (see Rudin (1981) for a case report). Although the present study has a cross-sectional design, preventing us from making any conclusions as to the causality of *ω*-3 FA intake and depression, it seemed unlikely that depression would change the intake of *ω*-3 FA. In the present study, no significant differences in total caloric intake and total fat intake were found between patients with depression and those without (Table 1). Our results suggest that low levels of *α*-LA or total *ω*-3 FA intake may contribute to a susceptibility to depression. A further clinical trial using supplements or a special diet is needed to clarify the independent effects of each *ω*-3 FA on depression.

*α*-Linolenic acid intake is known to have an influence on the fundamental structural and functional roles in the developing and mature nervous system (Bourre *et al*, 1993; Uauy *et al*, 1996), and an interaction between *α*-LA intake and neurotransmitters has been observed in animal experiments (Delion *et al*, 1996). *α*-Linolenic acid may influence depression via a mechanism that is different from that of DHA or EPA. Another possibility is that the total *ω*-3 FA (including *α*-LA) intake may reflect the concentration of DHA and EPA in the body or brain, rather than the sum of EPA and DHA intake only, since *α*-LA is the main source of *ω*-3 FA in the diet and can be converted into DHA or EPA in humans. Further investigation of a specific *ω*-3 FA biomarker is needed to confirm this speculation.

Our study has several limitations. First, a sampling bias was present in the data because all of the subjects attended a single institution and thus were not representative of lung cancer patients in general. Second, their possibility of a selection bias should be considered, since we did not have data on approximately 20% of the potentially eligible patients and the analysed subjects had a relatively better PS, an earlier clinical stage, and were younger than the nonanalysed subjects. Third, since only lung cancer patients who had been informed of their diagnosis but had not yet started their cancer treatment were included in the study, the results may not be applicable to other cancer patients and other cancer settings. Fourth, we used a self-administered questionnaire to evaluate *ω*-3 FA intake because a large sample of patients was needed for The Lung Cancer Database Project. The data obtained from the FFQ may not reflect the actual *ω*-3 FA intake as accurately as other more detailed methods, such as dietary records. Fifth, we did not use a structured psychiatric interview in this study (such as the Structured Clinical Interview for DSM-IV, a widely recognized standard). However, HADS has been shown to be reliable and valid for the screening of depression in patients with cancer (Razavi *et al*, 1990; Hopwood *et al*, 1991), and is widely used in the field of oncology (Patrick *et al*, 2003). Finally, a causal relationship between *ω*-3 FA intake and depression cannot be confirmed, because of the cross-sectional design of this study. To clarify the independent effects of each *ω*-3 FA on depression, a further clinical trial using supplements or a special diet is needed.

In conclusion, we did not observe a significant relationship between total EPA and DHA intake and depression in newly diagnosed Japanese patients with lung cancer, but significant inverse relationships between *α*-LA intake or total *ω*-3 FA intake and depression were noted. Our findings suggest that EPA and DHA may be of little use as an antidepressant in Japanese cancer patients. However, the association between *α*-LA intake or total *ω*-3 FA intake and depression should be further investigated. To elucidate the association between each *ω*-3 FA and depression in patients with cancer, further clinical research (including the examination of specific biomarkers) is needed.
